# Anemia-Related Knowledge and Practices Among Mid-adolescents in Government Schools of Nadia District, West Bengal: A Cross-Sectional Study

**DOI:** 10.7759/cureus.104694

**Published:** 2026-03-05

**Authors:** Poulami Bayen, Nandu Krishna, Jayita Pal, Manoj Ghosh, Shubham Vivekanand Chawale

**Affiliations:** 1 Public Health, Institute of Public Health Kalyani, Kalyani, IND; 2 Preventive and Social Medicine, Jawaharlal Institute of Postgraduate Medical Education and Research, Puducherry, IND; 3 Epidemiology, All India Institute of Hygiene and Public Health, Kolkata, IND; 4 Yoga, Centre for Integrative Medicine and Research, Manipal Academy of Higher Education, Manipal, IND; 5 Community Medicine, Lady Hardinge Medical College, New Delhi, IND

**Keywords:** adolescent health, anemia prevention, dietary practices, eastern india, iron-folic acid supplementation, maternal education, school-based survey

## Abstract

Background

Anemia is a major public health concern globally and in India, particularly among adolescents who are vulnerable due to rapid growth and increased nutritional requirements. Despite national programs such as Weekly Iron and Folic Acid Supplementation (WIFS), knowledge and preventive practices remain suboptimal. This study assessed anemia-related knowledge and practices among mid-adolescent (predominantly 14-16 years) school students and sociodemographic predictors of these outcomes.

Methods

A cross-sectional study was conducted among 464 students from selected government high schools in the municipal area of Nadia district, West Bengal, using a multistage random sampling approach. Data on sociodemographic characteristics, knowledge, and practices regarding anemia were collected using a locally developed questionnaire. Records of dietary intake, hygiene, and preventive services were reviewed. Data were analyzed using Jamovi software (v 2.6.26, jamovi project, Sydney, Australia). Descriptive statistics were summarized as frequencies and percentages, while multivariable logistic regression identified predictors of good knowledge and anemia-related practices.

Results

The mean age of participants was 14.7 ± 1.52 years, with 56% being male. The mean knowledge score was 3.61 ± 2.04 (Q1 = 3, median = 4, Q3 = 4), and more than half of the students (55.6%) demonstrated good knowledge of anemia. In multivariate analysis, class level (Class VIII: AOR 2.37; 95% CI 1.11-5.05) and family income showed a non-linear association with knowledge (<INR 20,000: AOR 2.30; 95% CI 1.08-4.88; >INR 70,000: AOR 4.26; 95% CI 1.20-15.08). The mean practice score was 6.20 ± 1.25 (Q1 = 5, median = 6, Q3 = 7), with 72.8% of students reporting good anemia-related practices, which were significantly associated with maternal education (≥middle school: AOR 1.65; 95% CI 1.10-2.46) and students’ knowledge (AOR 1.81; 95% CI 1.16-2.83). Despite these findings, notable gaps remained in deworming coverage (11%) and adherence to weekly iron-folic acid supplementation (42.5%).

Conclusions

Despite moderate awareness, comprehensive knowledge and preventive practices remain suboptimal. Strengthening structured school-based health education, improving iron-folic acid supplementation and deworming program delivery, and engaging mothers are key strategies to enhance anemia prevention among adolescents.

## Introduction

Anemia is a serious global public health problem among adolescents, characterized by reduced hemoglobin concentration or red blood cell mass that impairs tissue oxygenation, as defined by WHO criteria [1,2]. Although biological susceptibility varies by age, sex, and physiological state, the main mechanisms leading to anemia, such as nutrient deficiencies, impaired absorption, and increased red cell loss due to infections, are strongly influenced by individual knowledge and practices. Inadequate dietary intake, poor adherence to iron supplementation, delayed care seeking, and low uptake of preventive measures such as deworming directly disrupt erythropoiesis, underscoring the importance of knowledge and practice in anemia prevention and control [1-3].

Adolescence is a crucial growth period between 10 and 19 years of age, marked by rapid physical development and increased nutrient requirements, especially for iron, making this age group nutritionally vulnerable [3]. Anemia during adolescence can diminish cognitive performance, reduce school participation and productivity, and predispose girls to adverse reproductive outcomes later in life [3,4]. In India, anemia continues to be a major public health challenge among adolescents. According to the National Family Health Survey-5 (2019-21), approximately 59.1% of adolescent girls and about 31.1% of adolescent boys aged 15-19 years were anemic, reflecting a substantial gender disparity and a sustained burden [5]. A systematic review of community-based studies also estimated a pooled anemia prevalence of about 65.7% among adolescent girls in India, indicating pervasive undernutrition across settings [6].

Despite widespread recognition of the burden of anemia, evidence on knowledge and stated practices related to anemia, especially among mid-adolescent (14-16 years) government school students, remains limited, particularly in municipal areas [7-9]. Mid-adolescence represents a biologically vulnerable period characterized by rapid physical growth, increased iron requirements, and, among girls, the onset of menstruation. This stage also marks greater autonomy over dietary and health behaviors, making knowledge and practices particularly important for effective anemia prevention [7]. Municipal settings such as Kalyani in the Nadia district of West Bengal present a distinct socio-educational context, where adolescents experience a mix of urban amenities and persistent socio-economic vulnerabilities that may influence dietary habits, health awareness, and care-seeking differently from rural or metropolitan populations [5,6]. While previous studies have largely focused on estimating anemia prevalence and identifying biological and socio-demographic risk factors, few have explicitly examined adolescents’ knowledge and practices related to anemia prevention and control, particularly in municipal school settings [10-12].

Understanding these determinants is essential for designing targeted school-based health education and nutrition interventions. Even without measuring anemia prevalence, studying knowledge and practices is important because they are key determinants of anemia risk. Understanding gaps in awareness and preventive behaviors such as diet, iron and folic acid (IFA) intake, deworming, and hygiene helps identify targets for school-based and community interventions. This information guides effective health education, strengthens existing adolescent programs, and supports strategies to reduce future anemia burden. This study, therefore, aims to assess knowledge and practices related to anemia and identify their determinants among predominantly mid-adolescent school-going students in the Kalyani municipal area, offering context-specific insights for adolescent nutrition programming.

## Materials and methods

Study design and setting

A descriptive, school-based cross-sectional study was conducted among predominantly mid-adolescent school-going students in the Kalyani municipal area of Nadia district, West Bengal, India. The study was conducted over a six-month period, from February to July 2023, at the Institute of Public Health Kalyani, located in Kalyani, Nadia district, West Bengal. 

Study population

The study population comprised students enrolled in classes VIII to X in government high schools within the Kalyani municipal area, largely representing the mid-adolescent age group of 14-16 years. A few early and late adolescents were included because class-based sampling was adopted, and these students were studying in the same grades and exposed to similar academic and school health interventions; excluding them solely based on age would have been ethically inappropriate and operationally impractical.

Inclusion and exclusion criteria

Students studying in classes VIII to X and present during the data collection period were included after obtaining written informed consent from parents or guardians and assent from the students. Students who were chronically absent or sick at the time of data collection were excluded. Students in Class X, who were preparing for their board examinations and fulfilled the inclusion criteria, were included in the study because they were present at school for the submission of their project works. The survey was brief, taking only about 20 minutes to complete; therefore, participation did not meaningfully interfere with their academic activities.

Sample size estimation

The sample size was calculated using Cochran’s formula for a single population proportion, assuming a 95% confidence level, 5% absolute precision, and an expected prevalence of adequate knowledge regarding anemia of 35%, based on a study by Singh et al. (2019) [13]. After accounting for a 10% non-response rate, the final sample size was determined to be 385 students. This school-based study used a participatory approach, with prior permission from school authorities and class-wise sensitization of students. More eligible students were present and consented to participate than anticipated, so all were included to avoid exclusion and disruption of classroom activities. Consequently, the final sample size exceeded the minimum calculated requirement of 385, totaling 464 students. Including additional participants improved the precision and power of estimates without affecting study objectives, outcome definitions, or analytical methods. All analyses were conducted using the final sample size.

Sampling technique

A multistage sampling approach was employed to ensure representativeness. Of the seven government high schools in the study area, four schools were randomly selected, providing unbiased inclusion while covering a sufficient proportion of schools to reflect the diversity of the school population. Within each selected school, the number of students drawn from each class was determined using proportional allocation based on class-wise enrollment, and in schools with multiple sections, sections and students were randomly selected. This approach preserved the distribution of students across schools, classes, and sections, ensuring that the sample reflects the overall student population.

Study tool

Data were collected using a pretested, locally developed, semi-structured questionnaire validated through a comprehensive literature review, expert consultation, and field experience (Appendix 1). The questionnaire was translated into Bengali to ensure semantic equivalence and ease of comprehension among participants. Face and content validity were assessed by a panel of public health experts. A pilot study was conducted among 20 students in a school outside the study area to evaluate clarity, feasibility, and comprehension, and necessary modifications were made accordingly. Reliability of the instrument was assessed using the test-retest method over a two-week interval, demonstrating acceptable temporal stability (ICC = 0.78). The questionnaire was self-administered under supervision and comprised sections on sociodemographic characteristics, as well as items assessing knowledge and practices related to anemia. Responses were scored and categorized using predefined criteria for subsequent analysis.

Study variables

Socio-demographic variables included age, religion, class, parental education, father’s occupation, family type, family income, and dietary patterns. Knowledge variables assessed awareness of anemia, its causes, symptoms, and prevention. Practice variables captured self-reported behaviors, including hemoglobin testing, IFA tablet intake, regular consumption of vitamin C or B12-rich foods, deworming in the past year, and frequency of daily personal hygiene practices. Attempts to verify these responses using the school Weekly Iron and Folic Acid Supplementation (WIFS) registers or other records were not possible due to operational and administrative feasibility.

Operational definitions

Mid-adolescents

Students, primarily aged 14-16 years, correspond to the mid-adolescence [14]. However, to reflect the actual age distribution within the same school classes, students aged 12-13 years and 17 years who were enrolled in the same classes as the target group were also included and operationally classified as mid-adolescents for analytical purposes. This approach was adopted to maintain class-based sampling integrity and ensure representativeness of the school environment.

Poor Knowledge Regarding Anemia

Students who obtained a knowledge score below the predefined cutoff based on correct responses to questions assessing awareness of anemia, including its causes, symptoms, and preventive strategies. Knowledge scores were categorized using the median as the cutoff, with scores below it classified as poor knowledge.

Poor Anemia-Related Practices

Students who scored below the predefined cutoff on practice-related items assessing anemia-preventive behaviors, including dietary practices, iron-folic acid consumption, hemoglobin testing, deworming, and hygiene practices. Practice scores were categorized using the median as the cutoff, with scores below it classified as poor practice.

Parental Education

The highest level of parental education was classified as illiterate and below primary, primary (Class IV passed), middle (Class VIII passed), secondary (Class X passed), Higher Secondary (Class XII passed), graduate, and postgraduate and above.

Data collection procedure

After obtaining permission from the school authorities, the study objectives were explained to the students, and written assent was obtained from them along with written informed consent from their guardians. During questionnaire administration, selected students were seated separately to prevent discussion and peer influence, thereby minimizing response bias. The questionnaire was completed within 15-20 minutes under the supervision of the investigator, with assistance from teachers.

Data analysis

Data were entered into Microsoft Excel 2019 (Microsoft Corporation, Redmond, Washington, United States) and analyzed using Jamovi version 2.6.26 (jamovi project, Sydney, Australia). Descriptive statistics, including mean and standard deviation for continuous variables and proportions for categorical variables, were calculated. Responses to knowledge- and practice-related questions were scored to generate total knowledge and practice scores for each participant. Each correct response was awarded one point (+1), while incorrect responses received zero. For questions with multiple correct options, a score of one was assigned if a correct option was selected; otherwise, zero was assigned. Domain-wise scores were calculated by summing item scores, with possible scores ranging from 0 to 14 for knowledge and 0 to 16 for practices. Although all items were designed as single-best-answer multiple-choice questions, responses indicated partial knowledge for some items. Therefore, scoring was adapted to award one point for each correctly identified component, while incorrect responses received zero. Knowledge and practice scores were dichotomized into “good” and “poor” categories using the median as the cut-off. The median split, a simple distribution-based method, ensures balanced group sizes, avoids assumptions of normality, and is robust to outliers. Previous studies also used similar approaches [15]. Dichotomization was adopted to facilitate interpretation, enable identification of adolescents with relatively lower knowledge or practice levels, and support public health planning in school-based settings. Although this approach reduces score granularity compared with continuous analysis, it provides a clear and actionable distinction between groups. Associations between knowledge and socio-demographic variables, as well as between practice, socio-demographic characteristics, and knowledge levels, were examined using appropriate inferential tests. All analyses were two-tailed, with p-values <0.05 considered statistically significant. Multicollinearity among independent variables was assessed using variance inflation factors and tolerance values, and no significant multicollinearity was observed.

Quality assurance

Quality assurance measures included developing a standardized study protocol based on a thorough literature review, pre-testing the data collection tools to ensure clarity and appropriateness, and training and familiarizing investigators with field procedures. During data collection, adherence to the protocol was monitored, and supervisors cross-checked a subset of completed questionnaires for completeness and accuracy. Data were double-entered into Microsoft Excel 2019 and checked for inconsistencies and missing values before analysis. All steps were performed by trained investigators to maintain consistency, reliability, and validity of the study data.

Ethical considerations

Ethical approval was obtained from the Institutional Ethics Committee (IHFW/IEC/2820, dated: 13/04/2023). Permissions were obtained from school authorities prior to data collection. Participation was voluntary, anonymity was maintained, and no personal identifiers were recorded. Written assent was obtained from the students, and written informed consent was obtained from their guardians. No monetary compensation was provided, although refreshments were offered to participants.

## Results

The study included 464 mid-adolescent students, with a mean age of 14.7 ± 1.52 years. The majority of participants were aged 14-15 years (74.2%). Most students were enrolled in Classes VIII (36.2%) and IX (33.8%), and 56.0% were male. The majority were Hindu (94.4%) and lived in nuclear families (68.7%). Regarding parental education, 60.1% of fathers had secondary education, while 36.1% of mothers had completed middle school. Fathers’ occupations were primarily in business (39.0%) or farming (37.1%). The average family income was INR 22,789 ± 20,367 per month, with 65.9% of families earning less than INR 20,000 per month (Table 1).

**Table 1:** Socio-demographic characteristics of mid-adolescent students (N = 464)

Variable	Category	Frequency (%)
Class	VIII	168 (36.2)
	IX	157 (33.8)
	X	139 (30.0)
Gender	Male	260 (56.0)
	Female	204 (44.0)
Age (years)	12	1 (0.2)
	13	34 (7.3)
	14	171 (36.9)
	15	173 (37.3)
	16	77 (16.6)
	17	8 (1.7)
Religion	Hindu	438 (94.4)
	Muslim	19 (4.1)
	Others	7 (1.5)
Father’s Education	Illiterate & below primary	3 (0.6)
	Primary	36 (7.8)
	Middle	126 (27.2)
	Secondary	279 (60.1)
	Higher secondary	2 (0.4)
	Graduate	13 (2.8)
	Postgraduate & above	5 (1.1)
Mother’s Education	Illiterate and below primary	5 (1.1)
	Primary	43 (9.3)
	Middle	167 (36.1)
	Secondary	166 (35.9)
	Higher secondary	25 (5.4)
	Graduate	40 (8.6)
	Post-graduate & above	17 (3.7)
Father’s Occupation	Service	111 (23.9)
	Business	181 (39.0)
	Farmer	172 (37.1)
Family Type	Joint	142 (31.3)
	Nuclear	311 (68.7)
Family Income (INR/month)	<20,000	288 (65.9)
	20,000-50,000	92 (21.1)
	50,000-70,000	35 (8.0)
	>70,000	22 (5.0)

Although most students reported having heard about anemia, only about half could correctly define the condition, indicating a clear gap between general awareness and conceptual understanding. Knowledge regarding the causes of anemia was fragmented, with improper diet being the most commonly identified cause, while biological and medical factors such as worm infestation, chronic infections, and vitamin B12 deficiency were infrequently recognized.

Recognition of signs and symptoms was similarly incomplete. Pallor of the conjunctiva, palms, tongue, and nail beds was the most commonly identified symptom, whereas other clinical features, such as palpitations, shortness of breath, fatigue, and brittle nails, were less frequently mentioned. Knowledge regarding the prevention of anemia was largely limited to dietary measures, particularly the intake of iron-rich foods, while balanced dietary practices and the roles of protein and vitamin C were inadequately acknowledged.

Notably, no respondent was able to correctly identify all causes, signs, and symptoms, or preventive measures of anemia. This finding highlights a substantial deficiency in holistic understanding, despite relatively high levels of basic awareness (Table 2).

**Table 2:** Knowledge and practice regarding anemia among students (N = 464) The mean ± standard deviation of knowledge score was 3.61 ± 2.04 (1st quartile or Q1 = 3, median or Q2 = 4, 3rd quartile or Q3= 4), while the mean ± standard deviation of practice score was 6.20 ± 1.25 (Q1 = 5, Q2 = 6, Q3 = 7).

Variable	Category	Frequency (%)
Knowledge regarding anemia		
Heard about anemia	Yes	394 (85.7)
	No	66 (14.3)
Correct definition of anemia	Yes	238 (51.7)
	No	222 (48.3)
Correct knowledge of the causes of anemia	Intestinal worm infestation	1 (0.2)
	Chronic infection	7 (1.5)
	Vitamin B12 deficiency	16 (3.5)
	Iron deficiency	28 (6.1)
	Improper diet	298 (64.8)
	Don’t know	114 (24.6)
Correct knowledge of the signs and symptoms of anemia	Pale conjunctiva, palm, tongue, nail bed	311 (67.6)
	Palpitation, shortness of breath	40 (8.7)
	Fatigue	33 (7.2)
	Brittle nails	10 (2.2)
	Don’t know	67 (14.4)
Correct knowledge of the prevention of anemia	Protein-rich food	3 (0.7)
	Intake of iron- and vitamin C-rich food	56 (12.2)
	Intake of iron-rich food	335 (72.8)
	Don’t know	70 (15.1)
Practice regarding anemia		
Consumed iron-rich food	6-7 days	182 (43.4)
	3-4 days	141 (33.7)
	2-3 days	73 (17.4)
	None of the above	23 (5.5)
Consumed vitamin-rich food	6-7 days	176 (43.0)
	3-4 days	139 (34.0)
	2-3 days	70 (17.1)
	None of the above	24 (5.9)
Consumed protein-rich food	6-7 days	96 (35.7)
	3-4 days	91 (33.8)
	2-3 days	63 (23.4)
	None of the above	19 (7.1)
Handwashing with soap before food	Always	356 (76.7)
	Sometimes	64 (13.8)
	Never	44 (9.5)
Handwashing with soap after defecation	Always	395 (85.1)
	Sometimes	46 (9.9)
	Never	23 (4.9)
Hemoglobin checked (last year)	Yes	359 (77.4)
	No	105 (22.6)
Biannual deworming (last one year)	Yes	51 (11.0)
	No	413 (89.0)
Consumed iron and folic acid (IFA) tablets weekly	Yes	197 (42.5)
	No	267 (57.5)

Overall, dietary practices showed high reported consumption of iron- and vitamin-rich foods, with about two-fifths of participants consuming these foods on six to seven days per week. However, regular protein-rich food intake was comparatively lower, with only around one-third reporting consumption on six to seven days, indicating a potential gap in dietary quality.

Hygiene practices were generally satisfactory, as the majority of participants reported always washing their hands with soap before eating and after using the bathroom. Nevertheless, a small but notable proportion reported inconsistent or no handwashing, which may increase the risk of infections contributing to anemia.

Regarding preventive and screening practices, over three-fourths of participants had their hemoglobin checked in the last year, reflecting good coverage of anemia screening services. In contrast, biannual deworming coverage was very low, with less than one-fifth reporting receipt of deworming in the past year, highlighting a critical gap in anemia prevention efforts.

Weekly IFA supplementation adherence was suboptimal, with only 42.5% of participants reporting the use of IFA tablets on a weekly basis (Table 2).

Overall, more than half of the students (55.6%) had good knowledge of anemia, while 44.4% had poor knowledge. Good knowledge was highest among students in Class VIII (71.4%), in the younger age groups (12-14 years), and among those whose fathers were employed in service (64%) or had higher education. Statistically significant associations were observed for class (χ² = 26.8, p < 0.001), age (p < 0.001), and father’s occupation (χ² = 7.16, p = 0.028). Other factors, including parental education, religion, family type, and family income, showed trends but were not statistically significant (Table 3).

**Table 3:** Association of knowledge related to anemia with sociodemographic characteristics (N = 464) * = analysed using Fisher's exact test. All other parameters were analysed using the chi-square test.

Variable	Category	Good Knowledge n (%)	Poor Knowledge n (%)	χ² (df), p-value
Class	VIII	120 (71.4)	48 (28.6)	26.8 (2), <0.001
	IX	72 (45.9)	85 (54.1)	
	X	66 (47.5)	73 (52.5)	
Age (years)	12	1 (100.0)	0 (0.0)	<0.001*
	13	8 (23.5)	26 (76.5)	
	14	113 (66.1)	58 (33.9)	
	15	76 (43.9)	97 (56.1)	
	16	41 (53.2)	36 (46.8)	
	17	1 (12.5)	7 (87.5)	
Religion	Hindu	244 (55.7)	194 (44.3)	2.51 (2), 0.285
	Muslim	12 (63.2)	7 (36.8)	
	Others	2 (28.6)	5 (71.4)	
Father's Education	Illiterate & below primary	1 (33.3)	2 (66.7)	p = 0.074*
	Primary	25 (69.4)	11 (30.6)	
	Middle	64 (50.8)	62 (49.2)	
	Secondary	155 (55.6)	124 (44.4)	
	Higher secondary	0 (0.0)	2 (100.0)	
	Graduate	11 (84.6)	2 (15.4)	
	Post-graduate & above	2 (40.0)	3 (60.0)	
Mother's Education	Illiterate & below primary	1 (20.0)	4 (80.0)	11.5 (6), 0.075
	Primary	26 (60.5)	17 (39.5)	
	Middle	85 (50.9)	82 (49.1)	
	Secondary	90 (54.2)	76 (45.8)	
	Higher secondary	16 (64.0)	9 (36.0)	
	Graduate	30 (75.0)	10 (25.0)	
	Post-graduate & above	9 (52.9)	8 (47.1)	
Father's Occupation	Service	71 (64.0)	40 (36.0)	7.16 (2), 0.028
	Business	104 (57.5)	77 (42.5)	
	Farmer	83 (48.3)	89 (51.7)	
Family Type	Joint	81 (57.0)	61 (43.0)	0.0793 (1), 0.778
	Nuclear	173 (55.6)	138 (44.4)	
Family Income (INR/month)	<20,000	157 (54.5)	131 (45.5)	6.76 (3), 0.080
	20,000-50,000	53 (57.6)	39 (42.4)	
	50,000-70,000	15 (42.9)	20 (57.1)	
	>70,000	17 (77.3)	5 (22.7)	

The logistic regression model identified class and family income as significant predictors of good knowledge regarding anemia. Students in Class VIII had higher odds of good knowledge than those in Class X (AOR = 2.37, 95% CI 1.11-5.05, p = 0.026). Family income showed a non-linear association: students from low-income households (<INR 20,000/month) and high-income households (>INR 70,000/month) were more likely to have good knowledge than those in the lower-middle reference group (AOR = 2.30, 95% CI 1.08-4.88, p = 0.031; AOR = 4.26, 95% CI 1.20-15.08, p = 0.025, respectively). Father’s occupation (service) and the INR 20,000-50,000/month income group showed borderline significance (p = 0.089 and p = 0.058, respectively). Other factors, including age and parental education, were not significant in the adjusted model. The model was statistically significant (χ² = 43.4, p < 0.001) with moderate explanatory power (McFadden’s R² = 0.127) and fair predictive performance (overall accuracy: 61.2%, sensitivity: 62.7%, specificity: 59.5%) (Table 4).

**Table 4:** Predictors of good knowledge related to anemia among students (multivariable analysis, N = 464) Model fit: χ² = 43.4, df = 10, p < 0.001; McFadden’s R² = 0.127; Cox & Snell R² = 0.0723. Predictive performance: overall accuracy = 61.2%, sensitivity = 62.7%, specificity = 59.5%. Notes: Estimates represent log odds of “knowledge = Good” vs. “knowledge = Poor.” AOR: adjusted odds ratio; CI: confidence interval; Reference category indicated by “Ref”; * = median

Predictor	Category (Reference)	p-value	AOR (95% CI)
Class	VIII (Ref: X)	0.026	2.37 (1.11-5.05)
	IX (Ref: X)	0.441	0.81 (0.48-1.38)
Age (years)	Per year	0.4	0.86 (0.61-1.22)
Father’s Education	Middle school & above* (Ref: below middle)	0.259	0.76 (0.47-1.22)
Mother’s Education	Middle school & above* (Ref: below middle)	0.223	1.34 (0.84-2.14)
Father’s Occupation	Service (Ref: Farmer)	0.089	1.63 (0.93-2.86)
	Business (Ref: Farmer)	0.228	1.33 (0.84-2.11)
Family Income (INR)	<20000 (Ref)	-	1
	20000-50000	0.058	0.96 (0.48-1.91)
	50000-70000	0.031	0.43 (0.21-0.92)
	>70000	0.025	1.85 (1.08-3.17)

Overall, 72.8% of students reported anemia-related practices classified as “good,” although core preventive behaviors such as weekly IFA supplementation (42.5%), deworming (11%), and adequate protein intake (six to seven days/week) (35.7%) remained suboptimal, highlighting gaps in essential practices. In bivariate analysis, maternal education and student knowledge of anemia were significantly associated with good practices, while other sociodemographic factors showed no significant association. After adjusting for potential confounders in multivariable logistic regression, maternal education (≥middle school: AOR 1.65, 95% CI 1.10-2.46) and student knowledge (good vs poor: AOR 1.81, 95% CI 1.16-2.83) remained significant predictors. Other variables were not significant, and the model’s McFadden’s R² of 0.12 indicates modest predictive ability, suggesting that additional unmeasured factors may influence anemia-related practices. The model correctly classified approximately 72% of students, indicating reasonable overall predictive performance while underscoring the need for interventions that strengthen key preventive behaviors rather than relying on general practice scores alone (Table 5).

**Table 5:** Bivariate and multivariable analysis of predictors of good anemia-related practices (N = 464) AOR: adjusted odds ratio; CI: confidence interval; Reference category indicated by “Ref”; Model fit: McFadden’s R² = 0.12, overall classification accuracy = 72%; * = median

Variable	Category	Good Practice n (%)	Poor Practice n (%)	χ² (df), p-value	AOR	95% CI
Class	VIII	123 (73.2)	45 (26.8)	0.34 (2), 0.843	Ref	-
	IX	114 (72.6)	42 (27.4)		0.97	0.61-1.55
	X	98 (70.5)	41 (29.5)		0.91	0.56-1.49
Age (years)	<15*	150 (72.8)	56 (27.2)	0.04 (1), 0.842	Ref	-
	≥15	185 (72.0)	72 (28.0)		0.97	0.64-1.47
Gender	Male	189 (73.0)	70 (27.0)	0.11 (1), 0.737	Ref	-
	Female	146 (71.6)	58 (28.4)		0.95	0.63-1.43
Father’s Education	Below middle*	111 (67.7)	53 (32.3)	2.77 (1), 0.096	Ref	-
	Middle & above	224 (74.9)	75 (25.1)		1.32	0.87-2.01
Mother’s Education	Below middle*	144 (67.3)	70 (32.7)	4.99 (1), 0.026	Ref	-
	Middle & above	190 (76.6)	58 (23.4)		1.65	1.10-2.46
Father’s Occupation	Service	83 (74.8)	28 (25.2)	0.47 (2), 0.791	Ref	-
	Business	128 (71.1)	52 (28.9)		0.88	0.54-1.43
	Farmer	124 (72.1)	48 (27.9)		0.91	0.56-1.46
Family Type	Joint	106 (74.6)	36 (25.4)	0.66 (1), 0.418	Ref	-
	Nuclear	220 (71.0)	90 (29.0)		0.84	0.54-1.30
Family Income (INR/month)	<20000	207 (72.1)	80 (27.9)	1.19 (3), 0.755	Ref	-
	20000-50000	65 (70.7)	27 (29.3)		0.93	0.55-1.57
	50000-70000	26 (74.3)	9 (25.7)		1.07	0.50-2.27
	>70000	18 (81.8)	4 (18.2)		1.42	0.45-4.42
Knowledge of Anemia	Poor*	136 (66.3)	69 (33.7)	6.65 (1), 0.010	Ref	-
	Good	199 (76.3)	62 (23.7)		1.81	1.16-2.83

## Discussion

This study assessed knowledge and practices related to anemia among mid-adolescent school students and examined sociodemographic predictors influencing these outcomes. Anemia remains a major public health concern globally and nationally, particularly during adolescence, a critical period marked by rapid growth, increased nutritional requirements, and heightened vulnerability to micronutrient deficiencies [13,16,17]. Despite sustained programmatic efforts in India, the prevalence of anemia among adolescents remains high, underscoring the importance of understanding knowledge and practice gaps at the community level [5,6,18].

Although most students reported having heard of anemia, only about half could correctly define it, reflecting a common pattern in adolescent populations where awareness does not always translate into conceptual understanding [12,13]. Anemia was primarily attributed to poor diet, with limited recognition of its multifactorial causes, including infections, parasitic infestations, chronic inflammation, and micronutrient deficiencies [2,4,17]. Pallor was the most frequently identified symptom, while systemic signs such as fatigue, palpitations, and shortness of breath were poorly recognized, potentially delaying care-seeking and early diagnosis [13,17]. Similar gaps have been documented in other Indian and low- and middle-income country settings [8,19].

Higher knowledge levels among students in lower classes and younger adolescents (12-14 years) may result from more recent and frequent exposure to school-based health education, interactive teaching methods, and participation in health campaigns. In contrast, older students may receive less reinforcement, face greater academic pressures, and rely more on self-directed learning. The inclusion of two participants aged 12-13 years, slightly below the stated inclusion criterion, may have influenced age-stratified trends.

Notably, no significant gender differences in knowledge or practices were observed, suggesting relatively equitable access to school-based health messaging in this setting, despite known community-level disparities [12,13]. Overall, these findings highlight the need for comprehensive, symptom- and cause-based health education to improve understanding and preventive behaviours among adolescents.

Preventive knowledge was predominantly centered on iron-rich foods, with minimal acknowledgment of the role of vitamin C in enhancing iron absorption or protein in hemoglobin synthesis. This restricted dietary understanding aligns with findings from other adolescent studies in India and highlights gaps in nutrition education curricula [3,10,20]. Notably, none of the participants demonstrated comprehensive knowledge across causes, symptoms, and prevention, indicating a fragmented learning approach rather than a holistic understanding.

In contrast to limited knowledge, reported dietary and hygiene practices were relatively satisfactory. A substantial proportion of students reported frequently consuming iron- and vitamin-rich foods, as well as practicing regular handwashing. This apparent discordance between knowledge and practice has been described elsewhere and may be attributable to school-based nutrition programs, mid-day meals, parental influence, or social desirability bias in self-reporting [10,11,21]. However, regular consumption of protein-rich foods was comparatively lower, which is concerning given the essential role of protein in erythropoiesis and overall nutritional adequacy [3,17].

Preventive service utilization revealed important programmatic gaps. While hemoglobin testing coverage was relatively high, suggesting improved access to screening through school health initiatives, biannual deworming coverage was notably low. This is consistent with national and regional evidence indicating suboptimal implementation of deworming programs despite strong policy support and clear evidence of benefit [1,4,22]. Similarly, adherence to weekly iron folic acid supplementation was suboptimal, aligning with reports that side effects, inadequate counseling, and irregular supply reduce compliance under the WIFS and the Iron Plus Initiative programs [21].

Multivariate analysis suggests that students in lower classes had better knowledge, possibly reflecting more recent exposure to structured health education. The non-linear association with family income, where both lower- and higher-income groups had better knowledge than the middle-income reference group, may reflect differences in access to information, parental engagement, or school quality [5,19,20]. Father’s occupation showed borderline significance, indicating a potential influence of socio-occupational context on adolescent knowledge.

However, the reported “good knowledge” and “good practices” may be somewhat overstated, as notable gaps remain in understanding anemia’s multifactorial causes, symptom recognition, and adherence to preventive behaviors such as IFA supplementation and deworming, highlighting important lacunae in adolescent awareness and health practices.

Anemia-related practices were significantly associated with the mother’s education and the students’ knowledge levels. Adolescents whose mothers were educated at least up to middle school were more likely to report good practices, reinforcing evidence that maternal education plays a crucial role in shaping adolescent health behaviors [6,10]. Additionally, students with good knowledge were significantly more likely to demonstrate good practices, supporting the premise that improving understanding can positively influence behavior when enabling environments are present [12,18]. The findings indicate that higher maternal education is associated with better anemia-related practices among students, suggesting that mothers play a key role in shaping adolescents’ health behaviors. Engaging parents, particularly mothers, through school-based nutrition education, counseling during IFA supplementation, and awareness campaigns may reinforce healthy practices at home and enhance the effectiveness of anemia prevention programs among adolescents.

Overall, the findings indicate that higher maternal education and better student knowledge are associated with improved anemia-related practices, highlighting the potential influence of both parental guidance and adolescent awareness on health behaviors. Interestingly, students in higher classes (e.g., Class X) showed lower knowledge levels, possibly due to greater focus on board exams, reduced reinforcement of health education, and competing academic priorities. These results suggest that school-based, age-appropriate health education, regular reinforcement of anemia-related messaging, counseling during IFA supplementation and deworming campaigns, and active involvement of parents, particularly mothers, may help maintain knowledge and support better anemia-related practices among adolescents in India [23-29].

Future studies could build on these findings using longitudinal or intervention designs to explore the relationships between maternal education, adolescent knowledge, and anemia-related practices more rigorously. Including biochemical assessments of hemoglobin and micronutrient status would allow examination of the link between knowledge, practices, and actual anemia burden. Research could also assess out-of-school adolescents and home- or community-level influences, including parental involvement, dietary patterns, and peer effects, on anemia prevention behaviors.

Limitations

This study has several limitations. First, its cross-sectional design limits causal inference between sociodemographic factors, knowledge, and anemia-related practices. Second, information on dietary intake, hygiene behaviors, IFA consumption, hemoglobin testing, and deworming was self-reported and could not be verified due to administrative constraints, which may be subject to recall, social desirability, or reporting bias, potentially leading to overestimation of good practices. Third, the questionnaire was locally developed and pretested, including a test-retest reliability assessment, but it was not validated against an established instrument; therefore, the term “validated questionnaire” would overstate its rigor. Fourth, students who were chronically absent due to illness were excluded, an inherent limitation of school-based studies that may introduce selection bias. Fifth, the presence of teachers during data collection and the provision of school midday meals could have influenced responses and practice scores. Sixth, the study assessed knowledge and practices but did not include biochemical hemoglobin measurement, so the actual burden of anemia and its direct relationship with knowledge or practices could not be determined. Seventh, multiple bivariate comparisons were performed without formal correction for multiple testing, which may have increased the risk of Type I error; while subsequent binary logistic regression adjusted for confounders, the model’s McFadden’s R² was modest (0.127), indicating that additional unmeasured factors may influence practice outcomes. Finally, the study was conducted only among school-going adolescents, limiting generalizability to out-of-school adolescents, and some aspects of anemia-related knowledge, particularly less commonly recognized causes and preventive strategies, may not have been fully captured.

## Conclusions

This study highlights substantial gaps in comprehensive knowledge regarding anemia among mid-adolescent students, despite moderate awareness and decent hygiene and dietary practices. Critical preventive behaviors, including WIFS (42.5%) and annual deworming (11%), remain far below national targets, indicating that self-reported practices are mixed and programmatically inadequate rather than “generally satisfactory.” Knowledge was often incomplete, with a limited understanding of multifactorial causes, diverse symptoms, and preventive strategies. Older students exhibited relatively lower knowledge levels, likely reflecting academic pressures and reduced reinforcement of health education.

Sociodemographic factors, particularly class level, family income, and parental characteristics, influenced knowledge, while maternal education and students’ own knowledge were key determinants of anemia-related practices. Given that anemia disproportionately affects adolescent girls, these findings underscore the importance of gender-responsive programming, including integration of menstrual health education with anemia prevention efforts.

Overall, the results indicate an urgent need for structured, age-appropriate, and comprehensive school-based health education that goes beyond dietary messages to cover biological causes, symptom recognition, and preventive services. Strengthening counseling during IFA distribution and deworming, coupled with active parental engagement, particularly of mothers, can enhance adolescent health behaviors. Aligning these interventions with global frameworks such as WHO’s AA-HA! Guidance and the Lancet Commission on Adolescent Health can improve program relevance and support equitable, evidence-based anemia prevention strategies.

## Appendices

Appendix 1

Questionnaire

Section A: Knowledge Regarding Anemia

A1. Have you ever heard of anemia?

a.     Yes

b.     No

A2. Which of the following best describes anemia?

a.     A condition in which the blood has lower than normal hemoglobin

b.     A condition caused by germs in the blood

c.     High blood pressure

d.     None of the above

A3. What do you think are possible causes of anemia?

(Select all that apply)

¨ Iron deficiency

¨  Improper diet

¨ Intestinal worm infestation

¨ Chronic infection

¨ Vitamin B12 deficiency

¨  Don’t know

A4. Which of the following can be signs or symptoms of anemia?

(Select all that apply)

¨   Fatigue

¨  Pale conjunctiva, palm, tongue, nail bed

¨   Palpitation, shortness of breath

¨  Brittle nails

¨  Don’t know

A5. Which of the following can help prevent anemia?

(Select all that apply)

¨  Intake of iron-rich food

¨  Intake of iron and vitamin C-rich food

¨  Protein-rich food

¨   Don’t know

Section B: Dietary Practices

During the past 7 days, on how many days did you consume the following foods?

B1. Iron-rich foods (e.g., green leafy vegetables, jaggery, legumes, eggs/meat)

a.       2-3 days

b.      3-4 days

c.      5-6 days

d.     6-7 days

e.     None of the above

B2. Vitamin-rich foods (such as citrus fruits, amla, guava, tomatoes, meat, eggs, spinach, etc., for Vit-B12, Vit-C, folic acid)

a.       2-3 days

b.      3-4 days

c.      5-6 days

d.       6-7 days

e.      None of the above

B3. Protein-rich foods (such as pulses, milk, eggs, soy, nuts)

a.      2-3 days

b.      3-4 days

c.      5-6 days

d.       6-7 days

e.      None of the above

Section C: Hygiene Practices

C1. How often do you wash your hands with soap before eating food?

a.      Always

b.      Sometimes

c.      Never

C2. How often do you wash your hands with soap after using the toilet?

a.      Always

b.      Sometimes

c.      Never

Section D: Preventive Health Practices

D1. When was your hemoglobin level last checked?

a.      Within the past 12 months

b.      More than 12 months ago

c.      Never checked

d.      Don’t remember

D2. How many times have you had a deworming tablet in the last year?

a.      Twice

b.      Don’t remember

c.      Never

D3. Do you consume Iron and Folic Acid (IFA) tablets weekly?

a.      Yes

b.     No
